# Agreement of Three Posturographic Force Plates in the Assessment of Postural Stability

**DOI:** 10.3390/ijerph17093188

**Published:** 2020-05-04

**Authors:** Piotr Matłosz, Justyna Wyszyńska, Justyna Podgórska-Bednarz, Justyna Leszczak, Maciej Rachwał, Krzysztof Przednowek, Justyna Drzał-Grabiec, Katarzyna Walicka-Cupryś, Mariusz Drużbicki, Emilian Zadarko

**Affiliations:** 1 Institute of Physical Culture Sciences, Medical College of Rzeszów University, Rzeszów University, 35-959 Rzeszów, Podkarpacie, Poland; krzprz@ur.edu.pl (K.P.); bzidar@interia.pl (E.Z.); 2 Institute of Health Sciences, Medical College of Rzeszów University, Rzeszów University, 35-959 Rzeszów, Podkarpacie, Poland; jwyszynska@ur.edu.pl (J.W.); j.e.podgorska@gmail.com (J.P.-B.); leszczakjustyna.ur@gmail.com (J.L.); maciekrachwal@gmail.com (M.R.); justyna.drzal.grabiec@wp.pl (J.D.-G.); kwcuprys@o2.pl (K.W.-C.); mdruzb@ur.edu.pl (M.D.)

**Keywords:** balance, center of pressure, stability, static platform, sway

## Abstract

This study was designed to assess how the results obtained for three different posturographic platforms agreed with each other in an assessment of static postural stability. The study included 111 young healthy participants. A measurement of postural stability was made for each participant, with their eyes open and then closed, on each platform in a random order. The Romberg ratio, path length, and center of pressure (COP) area were analyzed. For all measures, significant differences (*p* < 0.05) were observed among the three force plates. The highest Spearman’s rank correlation was observed between Alfa vs. CQStab2P (0.20 to 0.38), and the lowest between Alfa vs. AccuGait (−0.19 to 0.09). Similar results were obtained for the concordance correlation coefficient (0.10 to 0.22 for Alfa vs. CQStab2P and −0.6 to 0.02 for Alfa vs. AccuGait). Bland-Altman analysis for values standardized (z-scores) against AccuGait indicated a low level of agreement between compared platforms, with the largest error between AccuGait vs. Alfa, and a slightly lower error between AccuGait vs. CQStab2P or Alfa vs. CQStab2P. The 95% limits of agreement ranged from 2.38 to 7.16 (Alfa vs. AccuGait), 2.09 to 5.69 (CQStab2P vs. AccuGait), and 1.39 to 7.44 (AccuGait vs. Alfa) in COP length with eyes open and COP length Romberg ratio, respectively. Special care is recommended when comparing values relating to COPs from different devices that are analyzed by different software. Moreover, unperturbed stance tests among young healthy adults can be questioned as a valid postural control parameter.

## 1. Introduction

An objective assessment of the balance system efficiency is very important for the evaluation of progress in motor development, therapy, rehabilitation, and sports training. Postural stability is a focal point of scientific areas such as kinesiology, biomechanics, neurology, sports medicine, sports training, rehabilitation, etc. [1,2,3]. In order to disseminate the results of analyses in these areas, studies should be standardized, using reliable measuring equipment to ensure reproducibility [4,5]. It is possible to maintain the right level of postural stability due to a complex feedback and anticipatory feed-forward system based on dynamic and precise processing and coordination of stimuli from the vestibule system, organ of vision, and proprioceptors [6,7,8,9]. Stimuli from the above-mentioned systems cause a reaction of the musculoskeletal system aimed at correcting body posture and avoiding loss of stability and falling [1,10,11]. In addition, when a standing person performs a quick movement or interacts with external objects leading to postural perturbations in known or unknown directions, the neuromuscular system adjusts through the implementation of two aspects of feed-forward postural control: anticipatory postural adjustments and anticipatory synergy adjustments [12,13,14]. Static posturography is among these methods, which contributes to the assessment of postural stability in an objective way. This method uses a platform that registers the pressure force and moments of the forces exerted by the standing person’s feet during the test. Based on the reading, the computer software of the platform calculates the coordinates of the center of pressure (COP) when the person is standing freely [15,16,17,18,19,20,21,22]. A stabilometric test is very useful for the assessment and detection of balance impairment and the risk of falling in elderly people or patients suffering from neurological disorders [1,9,10,11,18,20]. This is why an objective assessment of postural stability is of interest to an increasing number of researchers and clinicians. It is important to be able to compare the results of studies carried out in different laboratories using different equipment and software. For a long time, the reliability of postural stability measurements on force platforms was controversial and their results ambiguous [15,21,23]. There have been many studies on the validation and reproducibility of postural stability using force platforms, identifying the optimum conditions for such inquiries [5,16,17,19,22,24,25,26,27]. Nevertheless, there is a limited number of papers comparing static balance results obtained under the same conditions but using different equipment or software provided by manufacturers. One can find papers comparing two devices and identifying one of them as the gold standard to examine the concurrent validity of postural control measures [4,28]. According to the authors, there are no studies comparing static balance in a higher number of commonly used force platforms.

Therefore, the purpose of this study was to compare the results for three commonly used commercial posturographic platforms intended for clinical postural stability assessment under static conditions.

## 2. Materials and Methods

The study was conducted in accordance with the ethical rules of the Helsinki Declaration and approved by the Bioethics Committee at the Medical Department of the University of Rzeszów, (decision no. 6/05/2012). Participation in the study was voluntary and consent was obtained from participants prior to the study. All subjects were informed about the possibility of dropping out at any stage of the study. Neither patients nor the public were involved in the design or planning of this study.

### 2.1. Participants

**Figure 1:**
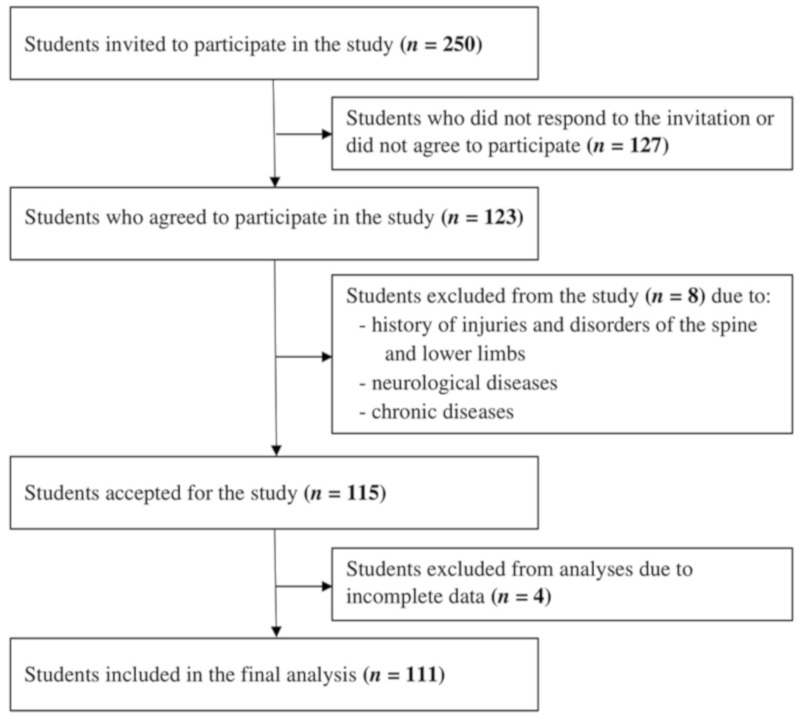
Study population selection procedure.

Volunteers (*n* = 111), recruited among students, were enrolled for the study (87 females and 24 males). The mean age of the participants upon enrolment for the study was 25.5 years (SD = 4.4 years). Their body mass index (BMI) values ranged from 16.3 to 31.1, while the majority of the studied population had a body size within the normal range (mean BMI = 22, SD = 3.6). Only healthy people were eligible for the study and the exclusion criteria included a history of injuries and disorders of the spine, pelvic girdle and lower limbs, spinalgia, and neurological and chronic diseases (Figure 1). Following previous studies, the above inclusion criteria were implied to prevent affecting outcomes by possible differences in the efficiency of the postural control system that could occur in older individuals or those with some postural issues [4,28].

### 2.2. Experimental Instrumentation

Three different devices were used to evaluate vertical ground reaction forces: two stabilometric force platforms from Polish manufacturers (“Alfa” designed by AC International East Sp. z o.o., Knurów, Poland and “CQStab2P” in a bi-platform version developed by CQ Elektronik System, Czernica, Poland) and the dynamographic platform “AccuGait” from Advanced Mechanical Technology, Inc., Watertown, MA, USA (AMTI).

The CQStab2P stabilometric force platform uses two measurement plates that enable independent assessment of the body COP parameters, separately for the right and left limbs. Each of the plates comes with three tensometric sensors, which register the forces and their moments. The signals from the sensors are amplified and processed in an analogue-digital converter with a 12-bit resolution. Then, the signals are sent in a digital form to a control and communication module from where they are transmitted to the computer software. The dimensions of each platform are 280 × 350 × 50 mm, and their total weight is about 10 kg. The resolution of each tensometric sensor is +/− 100 g and has the load capacity of 40 kg, which sets a total of 120 kg per platform. The maximum sampling frequency is 800 Hz. For the purposes of the study, the sample rate was set at 120 Hz. Thus, the platform meets the requirements of the medical device Directive 93/42/EEC.

The ALFA stabilometric platform enables the assessment of forces and their moments. The latter are used as the basis for calculating the static and dynamic parameters of COP when a person is standing on a stable surface. The equipment dimensions are 550 × 550 × 80 mm, and weighs 27 kg, while its maximum static load is 150 kg. The platform is featured with a system of four force sensors recording the changes in the patient’s body position. After amplification, the information is processed into a relevant output signal and sent online to a computer program. The sample rate was set at 120 Hz.

The dimensions of the AccuGait balance platform are 502 × 502 × 45 mm. The platform weighs 11.36 kg and uses Hall effect sensors for accurate measurement of the COP with internal amplification of the signal, a 100 Hz third-order analogue filter, and digital data transmission (32-bit floating point data containing six measurement channels). The data sampling frequency can be adjusted by the user within the range of 50–200 Hz. For the purposes of the study, the sample rate was set at 120 Hz. The sensors register forces and moments under static and dynamic conditions. The platform features the NetForce universal software.

Each of the platforms was connected to a dedicated computer with relevant software and valid controllers. To the best of our knowledge, there are no papers in which the reliability or validity of the analyzed devices has been assessed.

### 2.3. Procedures

Despite a high number of parameters referring to COPs generated by the software of the compared platforms, only two variables were calculated in a similar way by all analyzed platforms. Hence, the comparison included the length of the COP path and the area covered by the path records. The literature analysis confirmed that these parameters provided a very good assessment of body posture and are often used for this purpose [4,16,21,26,29,30]. In order to obtain clearer data, the authors standardized the units in which the results were quoted (mm for the path length and mm^2^ for the path area covered).

For all participants, the study was performed in the morning, in a quiet, bright, well-ventilated room. All platforms were placed on a stable surface (ceramic tiles) and then calibrated according to the manufacturer’s instructions. The study on the platforms was preceded by the participant resting in a sedentary position for five minutes. Participants undertook Romberg’s test on each device, standing freely on both legs with eyes open, and directly after the test with eyes closed. Each test lasted for 30 s, and was preceded by 10 s adjustment time before starting the recording. The participants were standing barefoot, wearing comfortable clothes [31]. The selected duration was a compromise between striving to reach maximum reproducibility of the results and preventing fatigue or discouraging the participants [17,22,24,26].

During the measurement with one’s eyes open, the test participant was not able to observe the COP position on the screen. Then, the study participant proceeded to another, random platform (to eliminate the effect of motor learning), where s/he performed an identical test. In order to ensure maximum reproducibility of the results, the study participants were instructed to stand barefoot on a thin cellulose sheet, orienting their feet freely and naturally, with both hands hanging loose along the trunk. The outline of the study participant’s feet was drawn on the cellulose sheet. The cellulose sheet with the outline of the feet was placed on each of the analyzed force platforms to ensure identical arrangement of the feet during each test (the CQStab2P platform plates were placed at a 4 cm distance from one another). For each person and on each platform, the test was performed according to the same oral instructions. The study participants were instructed to maintain the most stable body posture and look straight ahead at a point located 2 m away, on the opposite wall. If any type of unnatural behavior or distraction was noted during the test, the test for that person was repeated.

### 2.4. Data Analysis

The aim of the study was to compare the results obtained from commercial posturographic platforms in conditions most similar to clinical use. Clinicians working with patients usually need to obtain results immediately after testing without any post-processing analysis. In order to meet these needs, some manufacturers minimize the required setting of some important parameters by proposing some default settings (such as filtering, sampling frequency, etc.).

The sampling frequency was set at 120 Hz for all platforms [26]. Each software program provided the results instantly with data filtering built in the device or software filter solutions provided by the manufacturer without any post-processing analysis after the tests. Based on the results obtained from tests performed with eyes open and closed, the Romberg ratio was calculated for each participant on each device according to the following formula: ((EC − EO)/(EC + EO) × 100) [32,33].

The data were presented for six subsequent parameters:

1. COP area (mm2) eyes open (A-EO).
2. COP area (mm2) eyes closed (A-EC).
3. COP area Romberg ratio (A-RR).
4. COP path length (mm) eyes open (L-EO).
5. COP path length (mm) eyes closed (L-EC).
6. COP path length Romberg ratio (L-RR).

All statistical analyses were conducted using the GNU R Software environment for statistical computing (version 3.5.3), R Foundation for Statistical Computing, Vienna, Austria. Mean and standard deviation (SD) were used to describe the six variables. Correlation of the distribution of the variables with the normal distribution was verified with the Shapiro-Wilk test for all data. Two-way mixed intraclass correlation coefficients for mean (ICC_3_) measures were calculated to verify whether the results for the platforms were correlated. The concordance correlation coefficient (CCC) was also calculated to check the agreement between each pair of platforms. The correlation was also checked using Spearman’s rank correlation test. The differences between measurements performed on the analyzed platforms were assessed for each participant using the Friedman analysis of variance (ANOVA) test. In order to check which platforms provided significantly different results from one another, post-hoc analysis was performed using the Nemenyi multiple comparison test. The Wilcoxon matched pair test was used for an analysis of the relationships between individual results for different platforms, and the relationships were checked in pairs. All parameter values obtained from the compared platforms were also standardized against AccuGait platform results by z-scores, calculated by subtracting each parameter value from the mean AccuGait results and dividing the result by AccuGait standard deviations. The agreement between standardized values (z-scores) of all parameters obtained from the compared platforms was assessed using the Bland-Altman approach [34]. The statistical significance level for all analyses was assumed as *p* < 0.05.

## 3. Results

The mean (SD) results obtained for the compared platforms and ICC_3_ values are presented in Table 1 for the six analyzed parameters. The obtained ICC_3_ values were low for all of the analyzed parameters (all below ICC_3_ = 0.3) and a statistically significant correlation was observed only for A-EO.

Based on Spearman’s rank correlation test, statistically significant relationships (*p* < 0.05) were observed only for the L-RR parameter for all compared platforms. Significant results were also observed across all analyzed parameters obtained from the Alfa and CQStab2P platforms; however, none of them exceeded R = 0.4 (including negative values for the L-RR parameter). The concordance correlation coefficient test also provided very low values of agreement between each pair of analyzed platforms for all of the analyzed parameters (only A-EO exceeded CCC = 0.2) (Table 2).

Table 3 presents the results of the Friedman test for the measurements obtained for the compared platforms. Based on this data, it can be concluded that there was a statistically significant difference between the mean values obtained in the measurements for the analyzed platforms. The Nemenyi multiple comparison post-hoc test indicates significantly different results obtained for the analyzed platforms for most of the analyzed parameters.

Table 4 presents the results of the Wilcoxon matched pair test (p test) assessing the occurrence of significant differences between the particular participant results for different platforms for each analyzed parameter. The results obtained for the analyzed platforms significantly varied between them for most of the analyzed parameters.

Bland-Altman analysis of agreement between standardized values (z-scores) obtained from the compared platforms indicated the widest limits of agreement for the L-RR parameter. Moreover, taking into account all of the analyzed parameters, the widest limits of agreement were observed between the Alfa and AccuGait platforms (Table 5). Bland-Altman plots are presented in the Supplementary Materials.

## 4. Discussion

The study was designed to compare the results obtained for three different devices intended for the measurement and calculation of basic parameters referring directly to the ability to maintain postural stability. The results of the statistical analysis revealed the presence of significant differences between the values of the analyzed parameters obtained for each platform, with wide limits of agreement assessed by the Bland-Altman approach. This study was based on quiet stance tests, which are not a challenge for the postural control system of young healthy subjects, thus the outcomes could be different when studying an older cohort or subjects with postural problems. In their paper, Rogind et al. compared two older platforms for the assessment of postural stability (the Chattecx Balance System and the Kistler 9861A force platform). They performed a series of identical tests on the two devices on the day the study began and repeated them 2–4 weeks later. The tests involved standing on both legs with one’s eyes open and closed, and a sharpened Romberg test (tandem stance). Spearman’s rank correlation analysis based on similar parameters revealed poor, but statistically significant relationships between the platforms in the first test and a much stronger correlation between the results obtained 2–4 weeks later [4]. Golriz et al. examined the correlation of simultaneous indications between the Midot Posture Scale Analyzer and the AMTI AccuGait platform also used in this study. Golriz et al., considered the latter as the gold standard. To that end, five tests were carried out on each of the platforms for 31 study participants. Then, the area covered by COP in mm^2^ was compared to the movement speed in mm/s. The study revealed low simultaneous correlation of the analyzed platforms [28].

The literature contains some studies on the validation of the results for force platforms suited for postural stability tests including papers concerning the platforms compared in this study [5,28,35]. There are no grounds to challenge the repeatability of the equipment results compared in this paper. Taking into account the obtained results, however, one should be very cautious when comparing the parameters referring to COP obtained for different platforms and software. One should keep in mind the results obtained by other researchers using different equipment and/or software. Limited data in the literature concerning the comparison of equipment intended for postural stability tests encouraged the authors to proceed with this study. The authors also did not find any results comparing the platforms chosen for the study. Therefore, it is difficult to refer to the results obtained by other authors investigating the subject.

The relatively short test duration (30 s), in light of recent reports, was a limitation of the study, as it may not ensure the highest repeatability level. The time span was conditioned by the total number of tests in the entire study, these could cause disturbances resulting from fatigue or discouragement of the study participants. Some authors have suggested that, in some cases, extending the recording time could just add “noise” due to fatigue or diminished attention [26,36]. This was also the reason why only two tests were performed on each platform (with eyes open and closed), which also affected the reproducibility of the measurement and should be considered as a further limitation of the study. In order to reduce this impact, the Romberg ratio was calculated for the test on each platform and was included in the statistical analysis. Another important limitation of the study was the lack of post-processing analysis after the tests, which may impact their results. The authors decided to use the commercial software provided by the manufacturers of the analyzed devices in order to compare results obtained in conditions most similar to clinical use, assuming that many of the clinicians working with patients normally use the dedicated software of a particular platform in order to get results immediately after testing. In future studies, the obtained raw data should also be analyzed using the same software and identical analysis techniques to check whether potential differences are a hardware or software issue. One of the major limitations of this study was applying only quiet stance tests, which were not a challenge for the postural control system of young healthy subjects. The redundancy of the postural control system in this case could explain the large variations from platform to platform. The inclusion of only young healthy subjects could also be considered as an additional limitation of the study. An older cohort or subjects with particular postural issues could present different outcomes and future validation research should involve the above-mentioned groups or include more challenging postural stability tasks. Among the significant strengths of this study was the relatively large sample, which included 111 participants, and the study procedure, which strove to reach maximum reproducibility of the results.

## 5. Conclusions

One should take special care when comparing values applied to the COP obtained for different devices and analyzed by different software. Popular platforms commonly used to assess postural stability, despite high reproducibility of measurements, can vary significantly with regard to the numerical values of the calculated parameters in young healthy adults during an unperturbed stance. On the other hand, large variations observed between the compared platforms could also be the result of applying a task with no challenges for the postural control system in healthy young subjects, which calls into question the unperturbed stance test as a valid postural control parameter for healthy individuals.

## Figures and Tables

**Table 1:** Two-way mixed intraclass correlation coefficients for measures for the compared platforms for all analyzed parameters.

Compared Platforms		Analyzed Parameters					
		A-EO	A-EC	A-RR	L-EO	L-EC	L-RR
AccuGait	mean (SD)	102 (68.4)	204.7 (121.1)	31.8 (25)	662.7 (110)	731.1 (120.1)	4.9 (4.5)
Alfa	mean (SD)	131.8 (76.6)	211.4 (128.7)	20.9 (25.4)	169.1 (66.5)	263.5 (119.6)	20.7 (14.9)
CQStab2P	mean (SD)	119.2 (81.6)	116.1 (86.3)	−1.3 (28)	173.2 (52.6)	190.6 (46.5)	5.2 (11.3)
ICC_3_		0.2800	0.1200	0.0053	0.0845	0.0801	0.0840
p		0.0220 *	0.2100	0.4800	0.2900	0.3000	0.2900

* The identified correlation coefficients wee significant at *p* < 0.05. A-EO: center of pressure area (mm^2^) with eyes open. A-EC: center of pressure area (mm^2^) with eyes closed. A-RR: center of pressure area (mm^2^) Romberg ratio. L-EO: center of pressure path length (mm) with eyes open. L-EC: center of pressure path length (mm) with eyes closed. L-RR: center of pressure path length (mm) Romberg ratio. ICC_3_: Two-way mixed intraclass correlation coefficients.

**Table 2:** Results of the correlation tests for the compared platforms for all of the analyzed parameters.

Compared Platforms	Analyzed Parameters					
	A-EO	A-EC	A-RR	L-EO	L-EC	L-RR
Spearman’s rank correlation						
AccuGait/Alfa	0.01	0.09	0.05	0.05	0.01	−0.19 *
AccuGait/CQStab2P	0.06	0.07	0.17	0.11	0.05	−0.23 *
Alfa/CQStab2P	0.32 *	0.38 *	0.20 *	0.26 *	0.33 *	0.24 *
Concordance correlation coefficient						
AccuGait/Alfa	0.02	−0.06	−0.03	0.00	−0.01	−0.04
AccuGait/CQStab2P	0.07	−0.03	−0.06	0.00	0.00	−0.14
Alfa/CQStab2P	0.22	0.17	0.10	0.16	0.14	0.11

* The identified correlation coefficients were significant at *p* < 0.05. A-EO: center of pressure area (mm^2^) with eyes open. A-EC: center of pressure area (mm^2^) with eyes closed. A-RR: center of pressure area (mm^2^) Romberg ratio. L-EO: center of pressure path length (mm) with eyes open. L-EC: center of pressure path length (mm) with eyes closed. L-RR: center of pressure path length (mm) Romberg ratio.

**Table 3:** Results of the Friedman analysis of variance (ANOVA) test for the analyzed platforms with the Nemenyi multiple comparison post-hoc test for all of the analyzed parameters.

Friedman ANOVA	Analyzed Parameters					
	A-EO	A-EC	A-RR	L-EO	L-EC	L-RR
chi^2^	11.261	52.829	58.541	166.86	176.23	83.099
p	0.0036	<0.0001 *	<0.0001 *	<0.0001 *	<0.0001 *	<0.0001 *
Compared platforms	**Nemenyi post-hoc test for the analyzed parameters**					
AccuGait/Alfa	0.0023 *	0.6200	0.0690	<0.0001 *	<0.0001 *	<0.0001 *
AccuGait/CQStab2P	0.2137	<0.0001 *	<0.0001 *	<0.0001 *	<0.0001 *	0.6200
Alfa/CQStab2P	0.2137	<0.0001 *	<0.0001 *	0.8200	0.00022 *	<0.0001 *

* The identified correlation coefficients were significant at *p* < 0.05. A-EO: center of pressure area (mm^2^) with eyes open. A-EC: center of pressure area (mm^2^) with eyes closed. A-RR: center of pressure area (mm^2^) Romberg ratio. L-EO: center of pressure path length (mm) with eyes open. L-EC: center of pressure path length (mm) with eyes closed. L-RR: center of pressure path length (mm) Romberg ratio.

**Table 4:** Results of the Wilcoxon matched pair test for the analyzed platforms for all analyzed parameters.

Compared Platforms	Analyzed Parameters					
	A-EO	A-EC	A-RR	L-EO	L-EC	L-RR
Wilcoxon matched pair test						
AccuGait/Alfa	0.0020 *	0.6400	0.0680	<0.0001 *	<0.0001 *	<0.0001 *
AccuGait/CQStab2P	0.0620	<0.0001 *	<0.0001 *	<0.0001 *	<0.0001 *	0.6100
Alfa/CQStab2P	0.0410 *	<0.0001 *	<0.0001 *	<0.0001 *	<0.0001 *	<0.0001 *

* The identified correlation coefficients were significant at *p* < 0.05. A-EO: center of pressure area (mm^2^) with eyes open. A-EC: center of pressure area (mm^2^) with eyes closed. A-RR: center of pressure area (mm^2^) Romberg ratio. L-EO: center of pressure path length (mm) with eyes open. L-EC: center of pressure path length (mm) with eyes closed. L-RR: center of pressure path length (mm) Romberg ratio.

**Table 5:** Results from the Bland-Altman analysis of agreement between standardized values (z-scores) obtained from the platforms for all of the analyzed parameters

Compared Platforms		Analyzed Parameters (z-Scores)					
		A-EO	A-EC	A-RR	L-EO	L-EC	L-RR
AccGait/Alfa	MD 95% LOA	−0.44 2.92	−0.09 3.01	0.44 2.85	4.51 2.38	3.88 2.95	−3.56 7.16
AccGait/CQStab2P	MD 95% LOA	−0.25 2.95	0.73 2.46	1.33 3.12	4.47 2.09	4.53 2.13	−0.07 5.69
Alfa/CQStab2P	MD 95% LOA	0.19 2.83	0.82 2.25	0.89 2.76	−0.04 1.39	0.65 2.04	3.48 7.44

A-EO: center of pressure area (mm2) with eyes open. A-EC: center of pressure area (mm2) with eyes closed. A-RR: center of pressure area (mm2) Romberg ratio. L-EO: center of pressure path length (mm) with eyes open. L-EC: center of pressure path length (mm) with eyes closed. L-RR: center of pressure path length (mm) Romberg ratio. MD: mean difference between z-scores from the compared platforms. 95% LOA: limits of agreement, this value can be added to or subtracted from MD to obtain the upper and lower limits, respectively.

## Supplementary Materials

The following are available online at https://www.mdpi.com/1660-4601/17/9/3188/s1, Figure S1: Bland-Altman Plots for AccuGait vs Alfa, Figure S2: Bland-Altman Plots for AccuGait vs CQStab2P, Figure S3: Bland-Altman Plots for Alfa vs CQStab2P.
