# Supplementary material for: Agreement of Three Posturographic Force Plates in the Assessment of Postural Stability
Source: Int J Environ Res Public Health. 2020 May 4;17(9):3188. doi: 10.3390/ijerph17093188 (PMC7246617; doi:10.3390/ijerph17093188)
Supplement: Supplementary file 1 [file ijerph-17-03188-s001.zip › Figure S3. Bland-Altman Plots for Alfa vs CQStab2P.pdf]

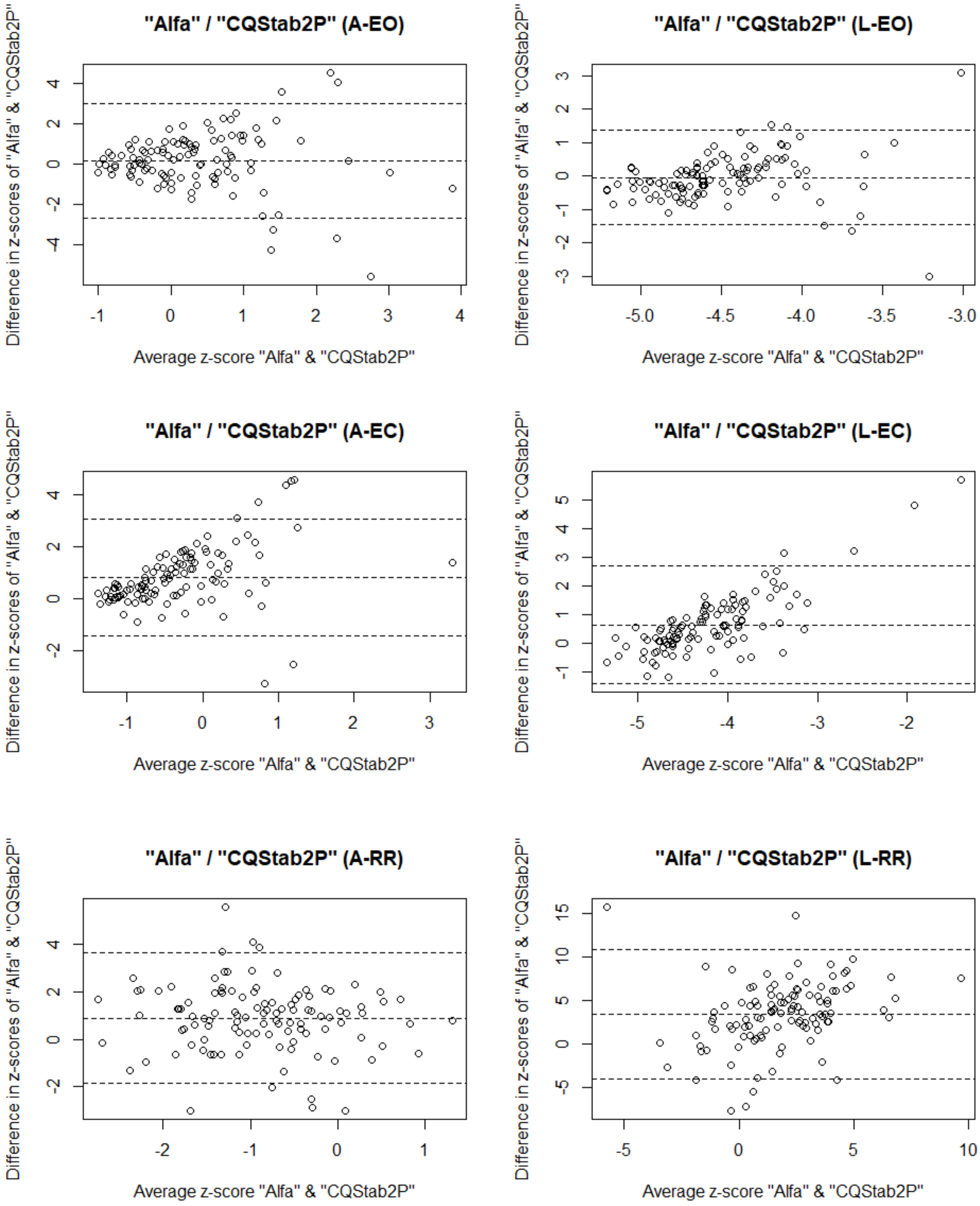

Figure S3. Bland-Altman plots representing comparison between values standardized (z-scores) against "AccuGait" platform obtained from "Alfa" and "CQStab2P" platforms. The difference between z-scores obtained from each two platforms is plotted against the average of these two variables. Horizontal lines indicating the mean differences and 95% limits of agreement corresponding to ± 2 SD of differences are shown. A-EO—centre of pressure area [mm2] with eyes open. A-EC—centre of pressure area [mm2] with eyes closed. A-RR—centre of pressure area [mm2] Romberg ratio. L-EO—centre of pressure path length [mm] with eyes open. L-EC—centre of pressure path length [mm] with eyes closed. L-RR—centre of pressure path length [mm] Romberg ratio.
